# Histone Demethylase KDM4C Stimulates the Proliferation of Prostate Cancer Cells via Activation of AKT and c-Myc

**DOI:** 10.3390/cancers11111785

**Published:** 2019-11-13

**Authors:** Ching-Yu Lin, Bi-Juan Wang, Bo-Chih Chen, Jen-Chih Tseng, Shih Sheng Jiang, Kelvin K. Tsai, Ying-Ying Shen, Chiou Hwa Yuh, Zong-Lin Sie, Wen-Ching Wang, Hsing-Jien Kung, Chih-Pin Chuu

**Affiliations:** 1 Institute of Cellular and System Medicine, National Health Research Institutes, Miaoli 35053, Taiwan; cylin071@nhri.org.tw (C.-Y.L.); bijuanwang@nhri.org.tw (B.-J.W.); Kimchen1026@gmail.com (B.-C.C.); mark0918@nhri.org.tw (J.-C.T.); 2 Nation Institute of Cancer Research, National Health Research Institutes, Miaoli 35053, Taiwan; ssjiang@nhri.org.tw (S.S.J.);; 3 Graduate Institute of Clinical Medicine, Taipei Medical University, Taipei City 110, Taiwan; 4 Pathology Core Lab, National Health Research Institutes, Miaoli 35053, Taiwan; minaka47@yahoo.com.tw; 5 Institute of Molecular and Genomic Medicine, National Health Research Institutes, Miaoli 35053, Taiwan; chyuh@nhri.edu.tw (C.H.Y.); zonlins@gmail.com (Z.-L.S.); 6 Institute of Molecular & Cellular Biology, National Tsing Hua University, Hsinchu 300, Taiwan; wenching.wc@gmail.com; 7 Graduate Institute of Cancer Biology and Drug Discovery, Taipei Medical University, Taipei 110, Taiwan; hkung@tmu.edu.tw; 8 Graduate Program for Aging, China Medical University, Taichung 404, Taiwan; 9 Graduate Institute of Basic Medical Science, China Medical University, Taichung 404, Taiwan; 10 Biotechnology Center, National Chung Hsing University, Taichung 402, Taiwan

**Keywords:** KDM4C, prostate cancer, c-Myc, AKT, Micro-Western Array

## Abstract

Our three-dimensional organotypic culture revealed that human histone demethylase (KDM) 4C, a histone lysine demethylase, hindered the acini morphogenesis of RWPE-1 prostate cells, suggesting its potential oncogenic role. Knockdown (KD) of KDM4C suppressed cell proliferation, soft agar colony formation, and androgen receptor (AR) transcriptional activity in PCa cells as well as reduced tumor growth of human PCa cells in zebrafish xenotransplantation assay. Micro-Western array (MWA) analysis indicated that KD of KDM4C protein decreased the phosphorylation of AKT, c-Myc, AR, mTOR, PDK1, phospho-PDK1 S241, KDM8, and proteins involved in cell cycle regulators, while it increased the expression of PTEN. Fluorescent microscopy revealed that KDM4C co-localized with AR and c-Myc in the nuclei of PCa cells. Overexpression of either AKT or c-Myc rescued the suppressive effect of KDM4C KD on PCa cell proliferation. Echoing the above findings, the mRNA and protein expression of KDM4C was higher in human prostate tumor tissues as compared to adjacent normal prostate tissues, and higher KDM4C protein expression in prostate tumors correlated to higher protein expression level of AKT and c-Myc. In conclusion, KDM4C promotes the proliferation of PCa cells via activation of c-Myc and AKT.

## 1. Introduction

Epigenetic alterations have been shown to be involved in the development or progression of cancer via regulation of histone modifications. Abnormal regulations of gene writing, reading, and erasing of histone acetylation or methylation marks promote tumor proliferation [1]. Overexpression, alteration, or mutation of human histone demethylases (KDMs) has been found in many types of cancers [1,2]. Eight KDM families composed of 28 members have been identified [3,4]. The KDM4A–KDM4C subfamily is a ~120 kDa protein that consists of a conserved JmjC catalytic domain and the double Plant Homeodomain (PHD) and Tudor domains, while the unique member KDM4D is only half size due to the loss of the PHD and Tudor domains. The KDM4A–KDM4D members are capable of removing the methyl group from H3K9me3/me2 [4,5]. The KDM4A–4C members can modulate androgen receptor (AR)-mediated transcription [6,7]. The expression of KDM4C is higher in castration-resistant prostate cancer (CRPC) [8]. Although the role of KDM4C in PCa is not well understood, KDM4C has been reported to be an oncogene in other cancers. The gene level of KDM4C is higher in aggressive, basal-like breast cancers [9]. The KDM4C interacts with HIF-1α and is required for breast cancer progression [10]. In colon cancer cells, KDM4C regulates the sphere formation of these cells via regulation of Wnt and Notch signaling pathways [11]. Overexpression of KDM4C increases the MDM2 oncogene [12]. Interestingly, KDM4C is required for embryonic development. During evolution, KDM4C is the conserved target of Nanog and is the histone demethylase implicated in the epigenetic reprogramming during the early embryogenesis in undifferentiated embryonic stem cells [13]. Additionally, KDM4C interacts with Ash21 and depletion of KDM4C causes downregulation of the pluripotency genes *Nanog*, *Pou5f1*, *Sox2*, *Myc*, and *Klf4* in embryos [14,15].

The process of prostate acinar morphogenesis, including the structural and functional differentiation, can be recapitulated in vitro using three-dimensional (3D) organotypic cell culture models with Matrigel [16]. When embedded in reconstituted basement membrane (rBM), human papillomavirus 18 immortalized, non-tumorigenic RWPE-1 cells form acini of polarized epithelium with a distinct lumen when cultured on Matrigel show a distinct laminin basement membrane and express α6β1 integrins at their basal end [16]. At the functional level, in the presence of androgen, these prostate acinar cells express prostate specific antigen (PSA) [16]. In contrast, human prostatic cancer (PCa) cells, such as LNCaP, PC-3, and DU-145 cells, form large amorphous cell aggregates without any organization or lumen. Therefore, normal prostate cells can undergo acinar morphogenesis while tumorigenic cells lose this differentiation ability. We previously reported that genes associated with prostate acini morphogenesis are great prognostic biomarkers for prostate cancer (PCa) [17]. We hypothesize that KDM4C plays an essential role in regulation of prostate acini morphogenesis as well as proliferation of PCa cells. Therefore, we used 3D organotypic culture to determine if KDM4C regulates prostate acinar morphogenesis and then we applied Micro-Western Array (MWA) to investigate the underlying molecular mechanism. Micro-Western Array is a high-throughput antibody-based proteomics system [18] which is composed of a GeSim Nanoplotter arrayer, a GE multiphor, and a Licor Odyssey infra-red scanner. It allows for the detection of protein expression levels or phosphorylation status changes of 96–384 different antibodies in 6–15 samples simultaneously. Our study suggests that KDM4C is a novel oncoprotein in PCa, and that KDM4C promotes the proliferation of PCa cells via activation of c-Myc and AKT signaling.

## 2. Results

### 2.1. KDM4C Interferes with Prostate Acini Morphogenesis

**Figure 1:**
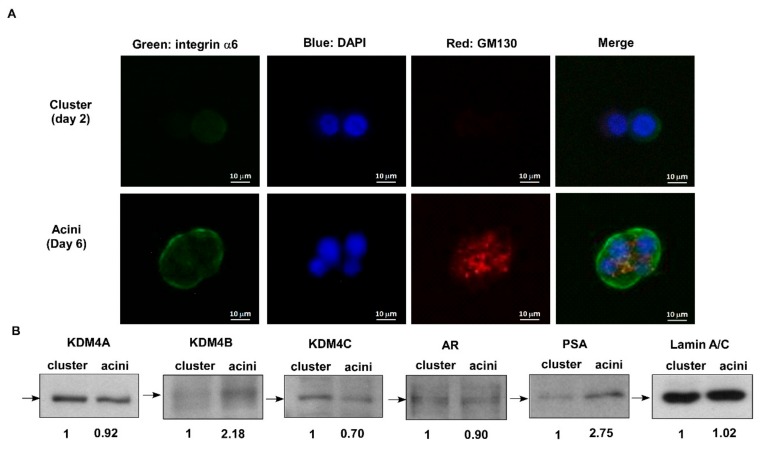
Decrease of human histone demethylase (KDM)4C expression during acinar morphogenesis of human RWPE-1 prostate epithelial cells. (**A**) Confocal images of RWPE-1 cells forming prostatic organoids in three-dimensional (3D) culture. The structures were immune-stained with basal extracellular membrane receptor α6-integrin (**green**) and the apical marker GM130 (**red**). Nuclei were counterstained with DAPI dye (**blue**). Scale bar = 10 μm. The RWPE-1 cells in 3D culture were collected on the 2nd day for cluster morphology and on the 6th day for acinar morphology for further protein expression analysis. (**B**) Protein expression of KDM4A, KDM4B, KDM4C, androgen receptor (AR), and prostate specific antigen (PSA) in cluster versus acinar morphology of RWPE-1 cells was determined by Western blotting. Lamin A/C was used as loading control.

**Figure 2:**
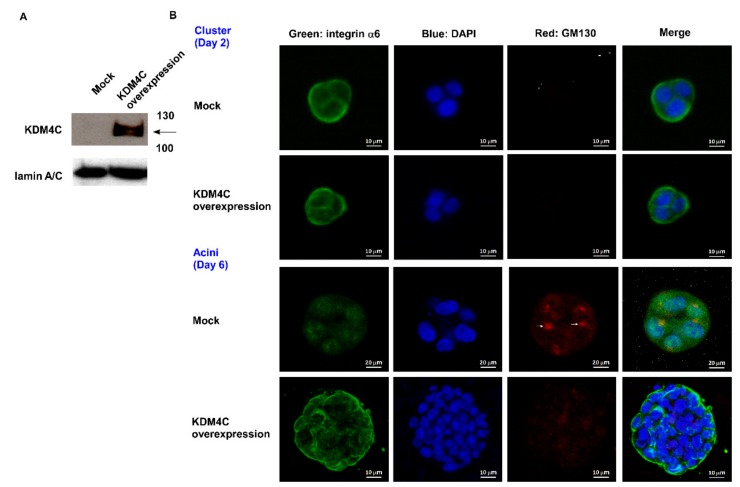
Overexpression of KDM4C hinders acinar morphogenesis of RWPE-1 cells. (**A**) Western blotting assay confirming the overexpression of KDM4C in RWPE-1 cells. Molecular weight label of 130 and 110 kDa was shown. (**B**) Confocal images of control RWPE-1 cells (**mock**) and RWPE-1 cells overexpressing KDM4C forming organoids in 3D culture. Images were taken on the 2nd and 6th day. The structures were immune-stained with basal extracellular membrane receptor α6-integrin (**green**) and the apical marker GM130 (**red**). Nuclei were counterstained with DAPI dye (**blue**).

The transformation of prostatic epithelium into prostate cancer (PCa) cells involves a gradual loss of cell adhesion and normal glandular structure [19]. Decrease of tissue-architecture-forming ability in prostate epithelial cells has been functionally linked to increased tumorigenicity toward PCa [20]. Previously, we used a gene array to demonstrate that genes involved in prostate glandular differentiation can serve as prognostic biomarkers for prostate cancer (PCa) [17]. A 3D culture of human prostate epithelial RWPE-1 cells in Matrigel allows for the cells to differentiate into a polarized acini structure, a procedure termed acinar morphogenesis. We reported that several genes with reduced expression levels during the acinar morphogenesis of prostate epithelial cells tended to be oncogenes [17]. As KDM4C is a histone demethylase implicated in epigenetic reprogramming during early embryogenesis in undifferentiated embryonic stem cells [13], we examined if KDM4C is involved in the regulation of prostate differentiation using a 3D organotypic culture assay with an rBM to recapitulate glandular-like tissues ex vivo [17]. A 3D culture of RWPE-1 cells in Matrigel caused the cells to differentiate into a polarized acini structure after 6 days (Figure 1A). The central part of the acini was hollow due to the apoptosis of inner cells. Compared to day 2, the protein levels of the prostate specific antigen (PSA) and KDM4B increased, while KDM4C decreased (Figure 1B). There was little change in the protein levels of KDM4A or the androgen receptor (AR). The increase in PSA expression confirmed the differentiation procedure of acinar morphogenesis [16]. According to our observation, we suspected that KDM4C can obstruct the prostate acinar morphogenesis. Indeed, overexpression of KDM4C (Figure 2A) hindered the formation of the acinar structure of RWPE-1 cells (Figure 2B). These observations revealed that elevation of KDM4C suppresses the differentiation of prostate cells and suggested that KDM4C is a potential oncoprotein in prostate cancer (PCa).

### 2.2. Knockdown of KDM4C Suppresses the Proliferation of Prostate Cancer Cells

**Figure 3:**
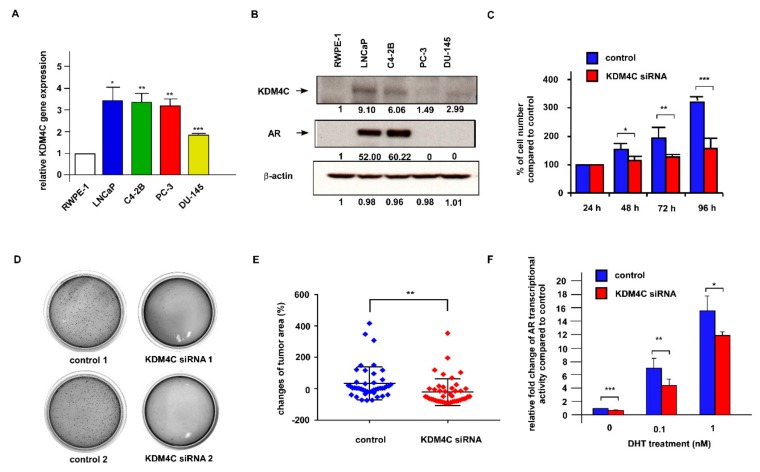
Knockdown of KDM4C in PCa cell lines suppressed cell proliferation and AR transcriptional activity. (**A**) Gene expression of KDM4C in RWPE-1, LNCaP, LNCaP C4-2B, PC-3, and DU-145 cells was analyzed by qRT-PCR. (**B**) Protein expression of KDM4C and AR in RWPE-1, LNCaP, LNCaP C4-2B, PC-3, and DU-145 cells was analyzed by Western blotting assay. β-actin was used as the loading control. (**C**) Proliferation of LNCaP C4-2B cells with or without KDM4C siRNA KD was determined by a 96 well Hoechst-dye proliferation assay for different time points (24, 48, 72, 96 h). Asterisks *, **, and *** represent statistical significance, *p* < 0.05, *p* < 0.01, and *p* < 0.001, respectively, among the two groups being compared. (**D**) Proliferation of LNCaP C4-2B cells with or without KDM4C KD cultured for 12 days was determined by a soft agar colony formation assay. All experiments were repeated at least three times and representative images of two different primers of siRNA knockdown are shown. (**E**) Tumor growth of LNCaP C4-2B xenografts with or without KDM4C KD in zebrafish after 24 h was quantified by calculating the area of tumor formation inside fish. Asterisks ** represent statistical significance, *p* < 0.01, among the two groups being compared. (**F**) Transcriptional activity of AR in LNCaP C4-2B cells with or without KDM4C KD was determined by reporter gene assay. LNCaP C4-2B cells were treated with increasing concentrations of dihydrotestosterone (DHT) for 24 h before the assay. Asterisks *, **, and *** represent statistical significance, *p* < 0.05, *p* < 0.01, and *p* < 0.001, respectively, among the two groups being compared.

To determine the oncogenic role of KDM4C in PCa cells, we examined the gene and protein expression levels of KDM4C in commonly used human PCa cell lines. Compared to normal human prostate epithelial cells in RWPE1, LNCaP FGC, LNCaP C4-2B, PC-3, and DU-145 PCa cells all expressed higher KDM4C mRNA (Figure 3A) and KDM4C protein levels (Figure 3B). To examine if KDM4C is involved in the regulation of PCa cell proliferation, we knocked down KDM4C in LNCaP C4-2B cells and LNCaP FGC cells. A Hoechst dye-based 96 well proliferation assay indicated that KDM4C knockdown (KD) suppressed the proliferation of LNCaP C4-2B cells (Figure 3C) and LNCaP FGC cells (Supplementary Materials Figure S1). Colony formation assay revealed that KDM4C KD retarded the colony formation of LNCaP C4-2B cells on soft agar (Figure 3D). Knockdown of KDM4C also reduced the tumor growth of LNCaP C4-2B cells in a zebrafish model (Figure 3E). These observations suggest that KDM4C is an oncoprotein in PCa cells. The KDM4C has previously been identified as an important AR co-regulator [21]. The reporter gene assay indicated that the KD of KDM4C reduced the transcriptional activity of AR under androgen treatment (Figure 3F), confirming that KDM4C is a co-activator of AR.

### 2.3. Knockdown of KDM4C Reduces Expression of Proteins Involved in AKT Signaling and c-Myc

**Figure 4:**
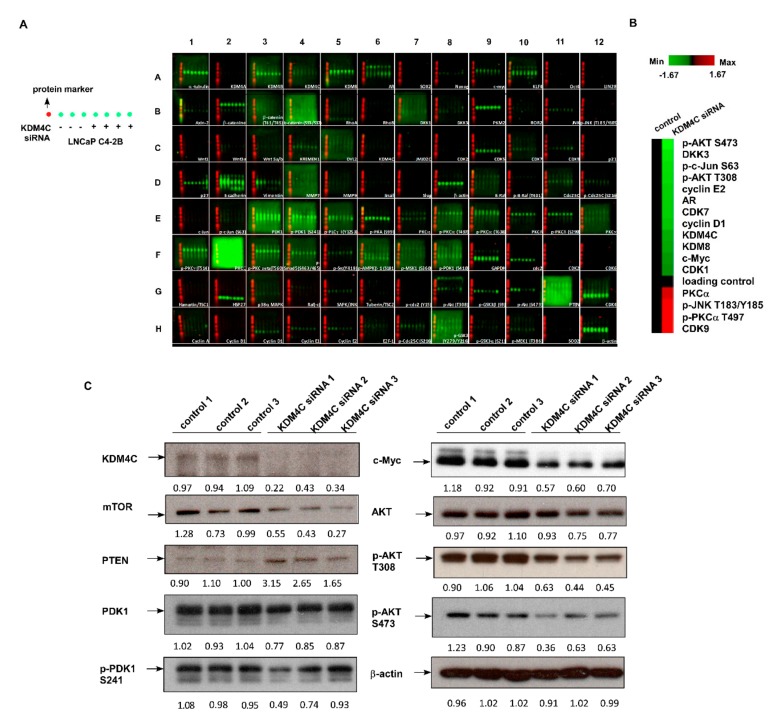
Micro-Western array (MWA) and Western blotting analysis indicated that knockdown of KDM4C suppressed AKT signaling and c-Myc in PCa cells. (**A**) Expression of proteins involved in cell cycle regulation, EGFR signaling, AKT signaling, Src signaling, and JNK signaling in LNCaP C4-2B cells with or without KDM4C siRNA KD was analyzed with MWA. (**B**) Expression level of proteins with a fold change larger than 2.0 or smaller than 0.5 between control and KDM4C knockdown analyzed from (**A**) is demonstrated in a heatmap with the value of the protein expression level being converted to log_2_. (**C**) Western blotting assay was used to confirm the change in proteins observed by MWA. Expression of KDM4C, mTOR, PTEN, PDK1, phospho-PDK1 S241, c-Myc, AKT, phospho-AKT T308 and phospho-AKT S473 was determined in triplicates of control LNCaP C4-2B cells and LNCaP C4-2B cells with KDM4C knockdown. β-Actin was used as the loading control.

To determine the molecular mechanism for how KDM4C regulates the proliferation of PCa cells, we performed the Micro-Western Array (MWA) analysis on 90 proteins involved in the regulation of cell cycle, cell proliferation, and cell survival (Figure 4A, Supplementary Materials Table S1). Compared to the control LNCaP C4-2B cells, LNCaP C4-2B cells with KDM4C KD expressed less phospho-AKT S473, phospho-AKT T308, DKK3, phospho-c-Jun S63, AR, c-Myc, CDK7, cyclin D1, KDM8, and CDK1, while KDM4C KD increased the protein expression of PKCα, phospho-PKCα, CDK9, and phospho-JNK T183/Y185 (Figure 4B). Conventional Western blotting further confirmed that KDM4C KD reduced the protein expression level of AKT, phospho-AKT T308, phospho-AKT S473, c-Myc, mTOR, PDK1, and phospho-PDK1 S241 but increased the abundance of PTEN protein (Figure 4C). These results suggest that KDM4C KD suppressed AKT signaling and c-Myc.

**Figure 5:**
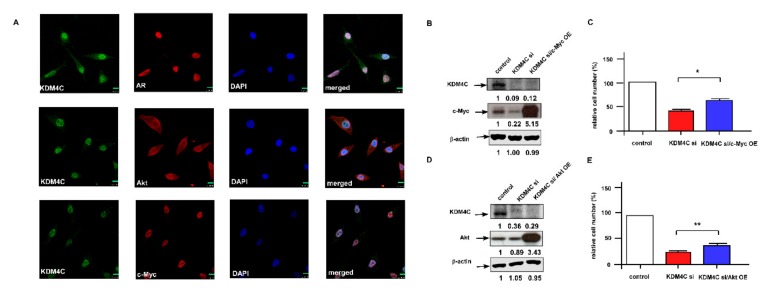
KDM4C, AR, and c-Myc co-localized in the nuclei of PCa cells and overexpression of c-Myc or AKT rescued the suppressive effect of KDM4C knockdown on the proliferation of PCa cells. (**A**) Immunofluorescent microscopy revealed that KDM4C co-localized with AR and c-Myc in the nuclei of LNCaP C4-2B cells. The green bar in each image represents 10 μm. (**B**) Western blotting confirmed the KDM4C KD and overexpression of c-Myc. (**C**) Proliferation of control LNCaP C4-2B cells, LNCaP C4-2B with KDM4C KD, and LNCaP C4-2B with KDM4C KD plus c-Myc overexpression was determined by a Hoechst-dye 96 well proliferation assay for 48 h. (**D**) Western blotting confirmed KDM4C KD and overexpression of AKT. (**E**) Proliferation of control C4-2B cells, C4-2B with KDM4C KD, and C4-2B with KDM4C KD plus AKT overexpression was determined by Hoechst-dye 96 well proliferation assay for 48 h. Asterisks * and ** represented statistical significance, *p* < 0.05 and *p* < 0.01, respectively, among the two groups being compared.

To investigate how KDM4C regulates AKT and c-Myc, we examined if KDM4C co-localizes with AKT or c-Myc in LNCaP C4-2B cells. As shown by the fluorescent microscopy images, KDM4C co-localized with AR and c-Myc in nuclei, while the distribution of AKT was mainly in cytoplasm (Figure 5A, Supplementary Figure S2A–D). Overexpression of c-Myc (Figure 5B,C) or overexpression of AKT (Figure 5D,E) rescued the proliferation inhibition of LNCaP C4-2B cells caused by KDM4C KD, suggesting that KDM4C promotes the proliferation of PCa cells via activation of AKT and c-Myc. Overexpression of AKT or c-Myc did not affect the protein expression of each other in KDM4C KD LNCaP C4-2B cells (data not shown), indicating that KDM4C regulates AKT and c-Myc parallelly. 

### 2.4. Prostate Tumors Express Higher Protein Levels of KDM4C, AKT, and c-Myc

To determine the relationship between KDM4C expression level and progression of disease in clinical PCa samples, we examined the KDM4C mRNA level in 24 normal prostate tissues and 86 primary tumors using quantitative real-time PCR with samples from the TissueScan Prostate Tissue qPCR Array (Supplementary Materials Figure S3). Compared to the normal prostate tissues, the KDM4C mRNA expression level was higher in prostate tumors. We further examined the expression levels of KDM4C mRNA in adjacent normal prostate tissues versus prostate carcinoma using Oncomine datasets, including the Arredouani PCa dataset (Supplementary Materials Figure S4A), Grasso PCa dataset (Supplementary Materials Figure S4B), Tomlins PCa dataset (Supplementary Materials Figure S4C), and Varambelly PCa dataset (Supplementary Materials Figure S4D). Among the 99 prostate tumor tissues and 55 adjacent normal prostate tissues analyzed, the mRNA expression level of KDM4C was higher in prostate carcinoma in all four datasets as compared to adjacent normal prostate tissues.

**Figure 6:**
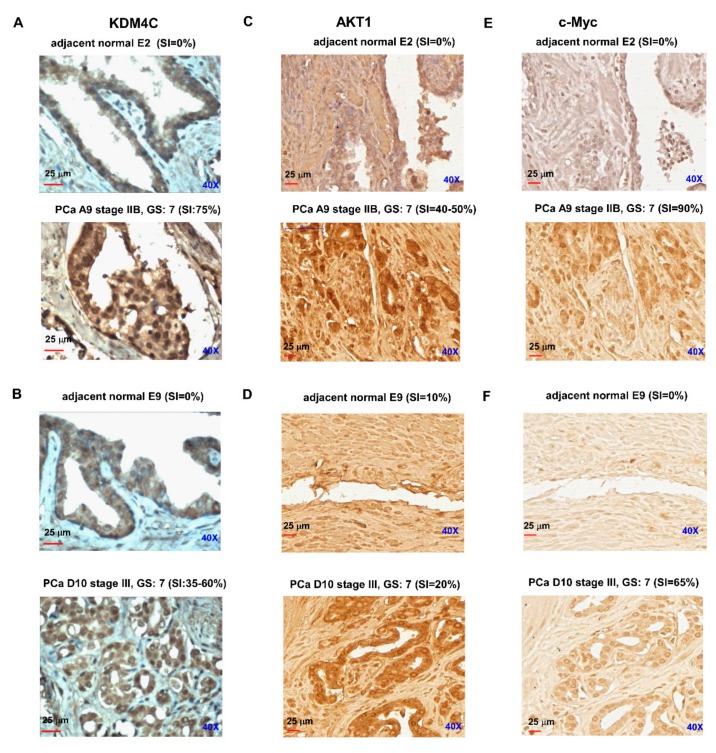
Protein expression level of KDM4C in prostate carcinoma versus adjacent normal prostate tissues. Protein expression levels of KDM4C, c-Myc, and AKT in prostate tumors in a commercial tissue array (CA4 tissue array from Super bio chips; 40 prostate tumors and 9 adjacent normal prostate tissues) were analyzed with IHC staining. Representative images of the protein expression levels of KDM4C (**A**,**B**), AKT1 (**C**,**D**), and c-Myc (**E**,**F**) in paired matched adjacent normal prostate tissues versus prostate tumors were demonstrated. The staining intensity of KDM4C protein, scale bar, and magnification of the microscopy were shown in each figure.

Finally, we determined the protein level of KDM4C in prostate tumors using immunohistochemical (IHC) staining. The IHC staining revealed that the intensity of KDM4C protein expression was much higher in prostate carcinoma as compared to paired adjacent normal prostate tissues (Figure 6A,B, Supplementary Materials Figure S5A,B). Interestingly, the protein abundance of AKT (Figure 6C,D) and c-Myc (Figure 6E,F) was also higher in prostate carcinoma tissues as compared to the paired adjacent normal prostate tissues. These observations suggested that in prostate tumors, expression of KDM4C is elevated and it is positively correlated to the expression of AKT and c-Myc.

## 3. Discussion

Three-dimensional culture of epithelial cells has provided a new avenue for studying biochemical and architectural signaling in cells in physiologically relevant contexts, recapitulating the developmental and pathological conditions such as tumorigenesis and providing novel mechanistic insights into tissue development. Three-dimensional scaffolds and extracellular matrices (ECMs) enable the formation of organotypic multicellular structures characterized by cell–cell and cell–matrix interactions, epithelial polarization, and differentiation [22]. Three-dimensional cell culture models involve the use of physiologically relevant ECM, such as collagens or laminin (Matrigel), and thereby can be used to mimic in vivo conditions to elucidate mechanisms of tissue development and cancer progression [23]. Invasive prostatic carcinomas and prostatic intraepithelial neoplasia (PIN) are characterized by a loss of normal cell organization, cell polarity as well as the adhesion between cell–cell and cell–basement membrane [16]. The process of prostate acinar morphogenesis, including structural and functional differentiation, can be reconstructed by 3D cell culture models [16]. The RWPE-1 cells, which are non-tumorigenic human cells immortalized with papillomavirus 18, are able to form polarized acini with a distinct lumen when cultured within rBM. Specifically, RWPE-1 acini show a distinct laminin basement membrane and express α6β1 integrins at their basal end [16,17]. In the presence of androgen, cells in RWPE-1 acini express prostate-specific antigen (PSA) [16]. By contrast, human PCa cells form large amorphous cell aggregates without any organization or lumen in 3D rBM. Therefore, normal prostate cells can undergo acinar morphogenesis while tumorigenic prostate cells lose this differentiation ability. We envisaged that the proteins antagonizing the prostate acinar morphogenetic process may play a role in prostate tumorigenesis and, thus, serve as potential oncoproteins. In this study, we demonstrated that the protein expression of the histone demethylase KDM4C decreased during the acinar morphogenesis of RWPE-1 cells and overexpression of KDM4C blocked acinar morphogenesis which implicate its potentially oncogenic role in PCa. It is interesting that expression of KDM4B protein increased during the acinar morphogenesis (Figure 1) which suggests the possibility that KDM4B may play an opposite role during prostate cell differentiation as compared to KDM4C. Further experiments will be needed to clarify the role of KDM4B in prostate development and differentiation.

**Figure 7:**
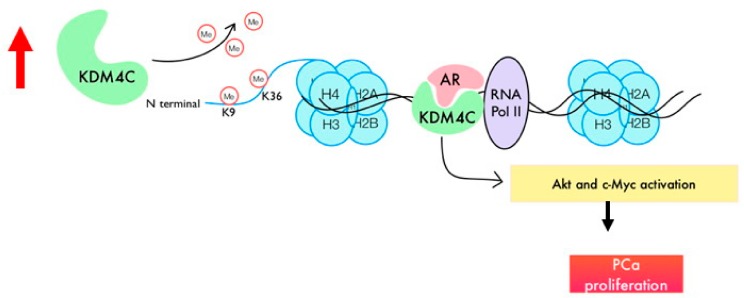
A schematic diagram for the role of KDM4C in regulation of PCa cell proliferation. Expression of histone demethylase KDM4C-targeting H3K9/ H3K36 is elevated in PCa cells, resulting in the activation of AKT signaling proteins and c-Myc and, thus, the stimulation of cell proliferation.

In this study, we showed that histone demethylase KDM4C promotes the proliferation of PCa cells via activation of c-Myc and AKT (Figure 7). Phosphatase and tensin homolog (PTEN) protein is a phosphatase dephosphorylating phosphatidylinositol (3, 4, 5)-trisphosphate. It is a negative regulator for the phosphoinositide 3-kinase (PI3K)-AKT signaling pathway [24]. Deletion of PTEN was observed in 40%–70% of PCa patients, resulting in activation of PI3K-AKT signaling. PI3K-AKT signaling plays an important role in the disease progression of PCa cells [25,26,27]. Elevation of PI3K-AKT signaling is associated with poor clinical outcome of PCa [25,28,29,30,31]. The level of phospho-AKT correlates with a higher Gleason score [32]. A high level of phosphorylated AKT1 is a strong predictor for prostate cancer recurrence [28]. The KDM4C has not previously been reported to regulate AKT. The c-Myc is an essential oncogene in PCa [33]. The mRNA level and nuclear protein level of c-Myc is elevated in human prostatic carcinoma and benign prostatic hyperplasia (BPH) [34,35]. The c-Myc stimulates the proliferation of PCa cells via direct binding with ribosomal DNA and activation of ribosomal RNA synthesis [36,37,38]. Our MWA result also revealed that KDM4C knockdown suppressed the expression of KDM8 (Figure 4). The KDM8 has been reported to be a coactivator of AR and plays an essential role during the progression towards CRPC [39]. The AR, KDM4C, and LSD1 have been reported to assemble on chromatin and work together to remove methyl groups and binding on androgen response element (ARE) to promote PCa proliferation [21]. The AKT, c-Myc, and AR are all essential for tumor growth and progression of PCa. As we discovered, KDM4C regulates the protein expression and phosphorylation of AKT and c-Myc as well as affects the AR transcriptional activity in PCa, and it is possible that KDM4C inhibitors may be beneficial for patients with PCa. A few KDM4 (JMJD2) inhibitors [40,41,42,43,44] or KDM4C-specific inhibitors [45] have been developed. Clinical trials will be needed to examine if treatment with a KDM4C inhibitor can inhibit AKT signaling and c-Myc activity and, therefore, suppress the tumor growth of advanced PCa in patient. It will also be important to determine if KDM4C inhibitors exhibit fewer undesired side effects as compared to AKT inhibitors or c-Myc inhibitors in PCa patients.

## 4. Materials and Methods 

### 4.1. Cell Culture 

The RWPE-1, LNCaP FGC, and PC-3 cells were purchased from Bioresource Collection and Research Center (Hsinchu City, Taiwan). The LNCaP C4-2B cell line was a gift from Hsing-Jien Kung (NHRI, Taiwan). The RWPE-1 cells were maintained in keratinocyte serum-free medium (Invitrogen, Carlsbad, CA, USA) supplemented with bovine pituitary extract, 10 ng/mL epidermal growth factor, and 0.5% penicillin–streptomycin (Invitrogen). The cells were embedded and grown within a thick layer of 3D rBM gel (Matrigel, BD Biosciences, San Jose, CA, USA) as previously described [46]. The PC-3 and LNCaP cells were maintained in DMEM (Gibco/Invitrogen, Carlsbad, CA, USA) supplemented with 10% fetal bovine serum (FBS; Atlas Biologicals, Fort Collins, CO, USA), penicillin (100 U/mL), and streptomycin (100 μg/mL). The LNCaP C4-2B cells were maintained in RPMI 1640 medium (Gibco/Invitrogen, Carlsbad, CA, USA) supplemented with 10% fetal bovine serum (FBS), penicillin (100 U/mL), and streptomycin (100 μg/mL).

### 4.2. The 3D Cell Culture

Non-tumorigenic prostate RWPE-1 cells (ATCC, Manassas, VA, USA) were cultured in keratinocyte serum-free medium (17005-042, Invitrogen, Carlsbad, CA, USA) supplemented with bovine pituitary extract, 10 ng/mL epidermal growth factor, and 0.5% penicillin–streptomycin [47]. The cells were embedded in reconstituted basement membrane (rBM) gel (B.D 356231, Matrigel, BD Biosciences, San Jose, CA, USA), collected, and assayed at day two cluster type and day six acini type as previously described [16,48].

### 4.3. Immuno-Fluorescent Staining and Confocal Image

The RWPE-1 cells were seeded at a density of 1 × 10^3^ in μ-slide (chambered coverslip) 8 well (80826, Ibidi, Bavaria, Germany) with rBM gel embedded. Whole culture immunofluorescent staining was performed as previously described [49]. Primary antibodies including rat anti-α6 integrin (CD49f) (MAB1378, Merck KGaA, Darmstadt, Germany), Alexa Fluor 647 mouse anti-GM130 (558712, BD Biosciences, San Jose, CA, USA), secondary antibody Alexa Fluor 488 goat anti-rat IgG (A11006, Invitrogen, Waltham, MA, USA), and cell nuclei were stained with DAPI (Invitrogen, Waltham, MA, USA). Images were captured by a confocal microscope (63 fold objective; TCS SP5 IIl; Leica, Wetzlar, Germany).

### 4.4. Cell Proliferation Assay 

The relative cell number was analyzed by measuring the DNA content of cell lysates with the fluorescent dye Hoechst 33,258 (Sigma, St. Louis, MO, USA) as described previously [50,51].

### 4.5. Knockdown of KDM4C with siRNA 

Human KDM4C siRNA (Human KDM4C ON-TARGET plus SMART pool) and randomly scrambled sequence control were purchased from Dharmacon. The transfection procedure was performed using lipofectamine RNAiMAX (Invitrogen, Carlsbad, CA, USA) according to the manufacturer’s recommended protocol. Cells were seeded at a density of 3 × 10^5^ cells/well in 2.5 mL complete medium in 6 well plates overnight. Forty newton meters 40 nM siRNA were used for control and KDM4C knockdown. Western blotting was used to confirm the knockdown of KDM4C. After KDM4C siRNA transfection via overnight incubation, LNCaP C4-2B cells were further transfected with c-Myc plasmid or AKT1 plasmid, both generous gifts from Shutsung Liao’s lab at the University of Chicago, as previously described [51]. The PLNCX2 plasmid and pcDNA3.1 plasmid were used as controls for c-Myc overexpression and AKT1 overexpression, respectively. PolyJet™ in vitro DNA transfection reagent was used for the transfection (SL100688, SigmaGen Laboratories, Rockville, MD, USA). 

### 4.6. Western Blotting Analysis 

Protein extracts were lysed in mammalian cell lysis buffer. Protein concentration was determined with the Bradford reagent (Bio-Rad Laboratories, Hercules, CA, USA) using a bovine serum albumin standard. Proteins were separated on 8%–10% SDS-PAGE gels. The antibodies detecting androgen receptor and c-Myc were purchased from Abcam (Abcam, Cambridge, UK). The antibody detecting KDM4B (JMJD2B) was purchased from Bethyl (Bethyl, TX, USA). The antibodies detecting AKT, Cdc2, Cyclin D1, Cyclin E1, GSK-3β, KDM4A (JMJD2A), phospho-AKT S473, phospho-AKT T308, phospho-Cdc2 Y15, and phospho-GSK 3β S9 were purchased from Cell Signaling Technology (Cell Signaling Technology, Danvers, MA, USA). Antibody detecting Cyclin E2 was purchased from Millipore (Burlington, MA, USA). The antibodies for β-actin and KDM4C were purchased from Novus (Novus, Littleton, CO, USA).

### 4.7. Quantitative Real-Time PCR 

The KDM4C mRNA level of human prostate tissue and different stages of prostate cancer were determined on TissueScan Prostate Tissue qPCR Array HPRT501~503 (OriGene Technologies, Rockville, MD, USA) according to the manufacturer’s instruction with Maxima SYBR Green/ROX qPCR Master Mix (2×) (Fermentas, Glen Burnie, MA, USA) and analyzed by ABI PRISM 7000 (Applied Biosystems/Life Technologies, Carlsbad, CA, USA). The mRNA expression level was normalized to β-actin.

### 4.8. AR Transcriptional Activity Assay

Control LNCaP C4-2B cells and C4-2B cells with KDM4C KD were co-transfected with 3xARE-luciferase plasmid and pRL-TK retina. After transfection, cells were pre-treated with dihydrotestosterone (DHT) for 24 h and then examined by ARE luciferase reporter assay. The AR transcriptional activity was measured by dual-luciferase reporter assay system (E-1910, Promega Corporation, Madison, WI, USA). Detailed procedures were performed according to the manual.

### 4.9. Micro-Western Arrays (MWAs)

Control LNCaP C4-2B cells and C4-2B cells with KDM4C KD were collected and lysed with MWA lysis buffer to lysis the cell. Lysates were boiled for 10 min, sonicated on the probe sonicator, and sheered with a 25 gauge needle 5 times. The MWAs were performed as previously described [18]. Blots were analyzed by Odyssey analysis software (Li-Cor Biosciences, Lincoln, NE, USA). Heatmaps were created using PermutMatrix software (LIRMM, Montpellier, France).

### 4.10. Tissue Array 

Protein expression levels of KDM4C in different stages of the prostate tumors were examined on commercial tissue arrays using IHC staining. The arrays were purchased from Super Bio Chips (CA4) (Seoul, Korea) which included 40 prostate tumors and 9 adjacent normal prostate tissues. Among them, there were 8 tumors of stage II and 32 tumors of stage III.

### 4.11. Statistical Analysis 

Data are presented as the mean +/− SD from three independent experiments. Student’s *t*-tests were used to evaluate the statistical significance of the results.

## 5. Conclusions

In conclusion, our observations suggest that KDM4C is a novel oncogene in PCa and that it interferes with prostate differentiation as well as stimulates the proliferation of PCa cells via activation of AKT signaling and c-Myc. Elevation of mRNA expression and protein level of KDMs have been detected in PCa tissues; therefore, KDMs may represent diagnostic tools or epigenetic targets for development of novel treatment against advanced PCa.

## Supplementary Materials

The following are available online at https://www.mdpi.com/2072-6694/11/11/1785/s1, Figure S1: Knockdown of KDM4C in PCa cell lines suppressed cell proliferation of LNCaP cells. Figure S2: Gene expression level of KDM4C mRNA in prostate carcinoma versus adjacent normal prostate tissues. Figure S3: Expression levels of KDM4C mRNA in prostate carcinoma versus adjacent normal prostate tissues from Oncomine datasets. Figure S4: Immunofluorescent microscopy analysis for localization of KDM4C with AR, c-Myc, and AKT. Figure S5: Protein expression level of KDM4C in prostate carcinoma versus adjacent normal prostate tissues. Table S1: Antibody list, Supplementary Material and Methods.
